# Gene Factors and Serotypes Related to Polysaccharide and Protein-Based Candidate Vaccines Among *Streptococcus agalactiae* Isolates

**DOI:** 10.3390/cimb48040399

**Published:** 2026-04-14

**Authors:** Vasil S. Boyanov, Alexandra S. Alexandrova, Raina T. Gergova

**Affiliations:** Department of Medical Microbiology, Medical Faculty, Medical University of Sofia, Zdrave Str. 2, 1431 Sofia, Bulgaria; alexandrova_sa@medfac.mu-sofia.bg

**Keywords:** vaccine, *Streptococcus agalactiae*, GBS, capsule, conjugated polysaccharide vaccine, protein-based vaccine

## Abstract

A new strategy to reduce the morbidity and mortality associated with invasive *Streptococcus agalactiae* (Streptococcus group B, GBS) diseases encompasses the development of vaccines. Candidate vaccines at different stages of clinical trials have been developed on capsular polysaccharides or protein antigens. We studied 328 GBS isolates identified using routine microbiological tests, latex-agglutination, and PCRs. The samples were categorised into two main groups: vaginal (69.2%) and extra-vaginal (30.8%). The molecular serotyping and target gene factors were determined using singleplex or multiplex PCRs. The most common serotypes identified were Ia (24.7%), V (22.0%), and III (18.9%). Serotypes I–V constituted a total of 89.0%. The non-typeable were 9.8%. The frequency of genes included in the recombinant GBS-NN (*rib* + *bca*) and GBS-NN2 (*epsilon + alp2/3*) vaccines were 54.3% and 40.8%. We noted a significant prevalence in the distribution of serotypes II, III, and non-typeable in GBS-NN, whereas serotypes Ia and IV were predominant in GBS-NN2. The serotype prevalence identified in our research was consistent with the data from our region and confirmed the predominance of the six main serotypes included in the hexavalent conjugated vaccine. We highlighted the importance of the combined administration of both protein vaccines, ensuring optimal vaccine coverage.

## 1. Introduction

*Streptococcus agalactiae* (Streptococcus group B, GBS) is an opportunistic pathogen. In newborns, infants, and elderly patients, it may cause severe, life-threatening infections [1,2]. GBS is a leading bacterial pathogen associated with neonatal infections, such as meningitis, sepsis, and pneumonia. The possible outcomes of these infections include abortion, early or late neonatal mortality, and neurological or sensory impairments in survivors. Neonatal infections manifest as early-onset disease (EOD, within 6 days after birth) or late-onset disease (LOD, within 7–89 days after birth) [3,4,5]. GBS is associated with infections of the urogenital tract in pregnant women, which can increase the risk of premature births. Furthermore, it is recognized as a common pathogen that causes invasive streptococcal diseases in non-pregnant adults, including skin and soft tissue infections, urogenital infections, bacteremia, along with rarer conditions such as pneumonia, meningitis, and endophthalmitis [6,7,8,9,10].

Preventive measures were established to reduce the morbidity and mortality associated with invasive GBS diseases. These strategies encompass screening, antibiotic prophylaxis, and the development of new promising approaches such as vaccines [11]. Several GBS vaccine candidates are currently under development and/or undergoing clinical trials, including the conjugated polysaccharide vaccines and the two recombinant alpha-like protein-based (Alp) vaccines GBS-NN and GBS-NN2. Computer models indicate that a vaccine exhibiting 80.0% efficacy and 90.0% coverage could reduce perinatal mortality rates significantly [12,13,14,15,16,17,18]. The primary target population for the GBS vaccine is pregnant women; however, it can be used for non-pregnant adults during outbreaks or in other very high-risk settings [19].

The capsular polysaccharide (CP) of GBS is considered as one of the most important virulence factors, helping to protect against the immune response and crucial for the survival of the bacteria. This is achieved by reducing the efficacy of phagocytosis, exhibiting antigenic mimicry, inhibiting the activation of the alternative complement pathway, and facilitating biofilm formation. There are 10 known serotypes (Ia, Ib, II–IX), classified according to the different chemical structure and antigenicity of the CP [14,20,21,22,23]. Initial trials were conducted with a monovalent and bivalent tetanus toxoid conjugate vaccine containing serotype III and serotypes II and III, respectively. Subsequent clinical trials tested trivalent (Ia, Ib, and III) and pentavalent vaccines (Ia, Ib, II, III, and V). The hexavalent vaccine conjugated to genetically detoxified diphtheria toxin (Ia, Ib, II, III, IV, and V) has been undergoing phase three clinical trials since 2023 [17,24,25].

The surface alpha-like protein (Alp) family includes Alp1 (Epsilon), Alp2, Alp3, Alp4, Rib and Alpha-C, encoded by genes *epsilon*, *alp2/3*, *alp4*, *rib*, and *bca*, respectively. Structurally, they consist of a C-domain (attached to the cell wall), an N-domain (extending beyond the capsule), and an intervening region with tandemly repeated sequences [18,26]. The recombinant model GBS-NN containing the fused N-domains of Rib and Alpha-C proteins exhibited protective immunity, with the amount of antibodies produced being inversely proportional to the risk of developing invasive diseases. Another potential candidate is the fused N-domains of Alp1, Alp2, and Alp3, referred to as GBS-NN2, which could be administered in combination with GBS-NN [15,16,18].

The aim of the study was to examine the components of conjugate and recombinant candidate vaccines, investigate the associations between them, and compare these findings with previous research conducted in our country. This would help in monitoring the most promising genetic determinants, crucial for GBS surveillance strategies and could contribute to the development of vaccines.

## 2. Materials and Methods

### 2.1. Specimen Collection

We examined 328 strains of GBS from outpatients and inpatients recovered during routine diagnostics. The samples were collected from two university hospitals located in Sofia city (University Multiprofile Hospital for Active Treatment (UMHAT) “Aleksandrovska” and UMHAT ‘Acibadem City Clinic Tokuda’) and one hospital in Pleven city (UMHAT “Georgi Stranski), Bulgaria, between 2021 and 2025. The study was approved by the Ethics Committee of the Medical University—Sofia, protocol code KENIMUS No 7/ 3 April 2026. We categorised the samples into two groups according to their source. The first group consists of vaginal samples (*n* = 227, 69.2%) collected from pregnant and non-pregnant women. The second group contains extra-vaginal samples (*n* = 101, 30.8%) divided according to their source into invasive materials (53.5%) and non-invasive samples (46.5%). Samples from vaginal secretions were collected from patients with genital infections, where GBS was isolated as a co-infectious agent in bacterial vaginosis or as a leading pathogen in aerobic vaginitis. The invasive materials were obtained from typically sterile locations, including soft tissue wound aspirates, tracheal aspirates, blood cultures, and ear fluid aspirates, whereas the non-invasive samples were derived from urine, sperm fluid, and eye discharge. In the extra-vaginal group, nearly half of the patients had urogenital infections, including cystitis, pyelitis, pyelonephritis, and prostatitis (45.5%), followed by skin and soft tissue infections (39.6%), pulmonary diseases (5.9%), infections in the ear and eye (5.0%), and bacteremia (4.0%).

### 2.2. Identification of GBS Strains and DNA Extraction

GBS was presumptively identified by colony and Gram staining morphology, the presence of β-hemolysis on blood agar, negative catalase and PYR tests, lack of susceptibility to bacitracin, and a positive test for CAMP. Serological confirmation was performed using the latex-agglutination test for the Lancefield B serological group (PathoDxtra Strep Grouping Kit ThermoScientific, Oxoid, Basingstoke, UK). If necessary, subsequent biochemical identification was performed with Crystal GP (Beckton Dickinson, Kelberg, Germany) or Phoenix™ M50 (Becton Dickinson, Franklin Lakes, NJ, USA).

DNA extractions from pure cultures of *S. agalactiae* were performed with the DNA-Sorb A DNA Extraction Kit (Sacace Biotechnologies Srl, Como, Italy) according to the manufacturer’s instructions. All DNA extracts were stored at −70 °C.

Following the DNA extraction, all strains were tested for the presence of the identification gene using STRA-AgI and II primers, previously described by Delannoy et al. (2013) [27]. The described primers target the 16S–23S rRNA intergenic spacer region. Although these primers were used in animal studies, they target highly conserved sequences, ensuring universal amplification in human and animal isolates [27,28,29].

### 2.3. Serotyping

Determination of GBS serotypes Ia, Ib, II–VII was conducted with a PCR amplification protocol containing two multiplex reactions using primers previously described by Poyart et al. (2007) [30]. First multiplex reaction (reaction 1) contained serotypes Ia–IV, while the second one (reaction 2)—serotypes V–VIII. The last serotype IX was examined as a third separate PCR amplification (reaction 3) using primers described by Imperi et al. (2010) [31] (Table S1). The reaction conditions were initial denaturation at 95 °C for 5 min, followed by 30 cycles consisting of denaturation at 95 °C for 30 s, annealing at 61 °C (reaction 1), 62 °C (reaction 2), and 60 °C (reaction 3) for 30 s, and elongation at 72 °C for 1 min; final elongation at 72 °C for 5–10 min.

### 2.4. PCR Amplification of the Gene Determinants of Alp

The Alp gene determinants were examined by PCR using primers shown in Table S1 [32,33,34]. The reaction conditions were initial denaturation at 95 °C for 5 min, followed by 30–35 cycles consisting of denaturation at 95 °C for 30 s, annealing at 58 °C (*bca*), 60 °C (*rib*), 61 °C (*epsilon*), and 61 °C (*alp 2/3*) for 30 s, and elongation at 72 °C for 1 min; final elongation at 72 °C for 5–10 min. The *alp4* was not examined due to its extreme rarity, as it is not reported in most studies and is not included in the candidate vaccines. Furthermore, the genes *alp2* and *alp3* possess identical sequences and were detected together with a single pair of primers [16,18].

For all described PCR reactions, we used prime Taq premix 2× (Genetbio Co. Ltd., Daejeon, Republic of Korea), and the results were read by gel electrophoresis using GelRed^®^ Nucleic Acid Gel Stain (Biotium, Inc., Fremont, CA, USA). *S. agalactiae* ATCC^®^ 27956™ and no-template controls were used in the molecular genetic experiments. Molecular weight markers were used to determine the resulting PCR products: 100–1000 bp DNA Ladder (Meridian Bioscience, Cincinnati, OH, USA) or 100–5000 bp DNA Ladder Extended (Carl Roth GmbH, Karlsruhe, Germany). The detection of PCR products was carried out using a recording and documentation system InGenius 3 (Syngene, Cambridge, UK). The lack of primer dimers or contamination in the no-template control, along with the detection of the expected band in the specified position across multiple independent experiments, validated the reliability of our findings. Although we did not conduct technical replicates of the control in the final dataset, all experimental samples, along with the negative and positive controls, were placed on the same gel and validated through electrophoresis.

### 2.5. Statistical Analysis

Statistical analyses were performed using IBM SPSS Statistics for Windows v19.0 (IBM Corp., Armonk, NY, USA) and R Statistical Software for Windows v4.5.2 (R Foundation for Statistical Computing, Vienna, Austria). Fisher’s exact test was used. A p-value indicates the unadjusted significance levels related to an individual test. The correction of multiple comparisons was applied using the Benjamini–Hochberg procedure to control for the false discovery rate (FDR). A q-value represents a *p*-value that has been adjusted [35]. Results were considered significant if q ≤ 0.05. For the difference between two proportions, 95% confidence interval (CI) that did not include zero indicated significant differences; for odds ratios (OR), an interval that did not include the null value of 1.0 was considered statistically significant. An overall association test between GBS serotypes and *alp* genes was performed using Fisher’s exact test with Monte Carlo simulation (based on 10,000 replicates).

## 3. Results

### 3.1. Studied Population

In the current study, 328 non-duplicate GBS strains, isolated during routine diagnostics, were included. The patients examined, both outpatient and inpatient, were aged between 0 and 93 years. In the vaginal materials, patients aged between 21 and 39 years predominated, with an average age of 36.4 years. Among them, pregnant women constituted 27.8%, while non-pregnant women were 72.2%. Regarding extragenital samples, patients aged between 41 and 69 years predominated, with an average age of 50.5 years.

### 3.2. Distribution of Capsular Serotypes Among the Studied Strains

**Figure 1:**
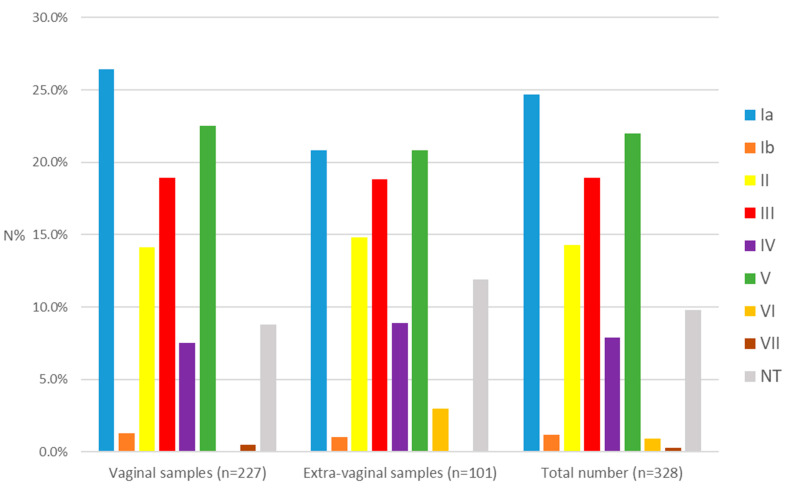
Distribution of serotypes according to the sample source. Ia, Ib, II–VII—serotypes identified in GBS. NT—non-typeable. N%—percentage of the total number within each group.

Among all the isolates examined, the most prevalent serotypes were Ia (24.7%), V (22.0%), and III (18.9%), followed by II (14.3%), IV (7.9%), Ib (1.2%), VI (0.9%), and VII (0.3%). The non-typeable were 32 (9.8%). Serotypes VIII and IX were not identified. Serotypes Ia, Ib, II, III, IV, and V (I–V) constituted a total of 89.0%, while VI and VII—1.2% (Figure 1). In the group of isolates from vaginal secretions, serotypes Ia (26.4%), V (22.5%), and III (18.9%) had the highest frequency. The same serotypes predominated in both subgroups—pregnant and non-pregnant. In the group of extragenital materials, the distribution was Ia (20.8%), V (20.8%), and III (18.8%). In the invasive group, the serotypes with the highest frequency were Ia (24.1%), III (20.4%), and II (20.4%), while in the non-invasive group—V (23.4%), Ia (17.0%), and III (17.0%). No statistical significance was found in the distribution of serotypes between the studied groups and subgroups regarding the sample source (q > 0.05).

**Figure 2:**
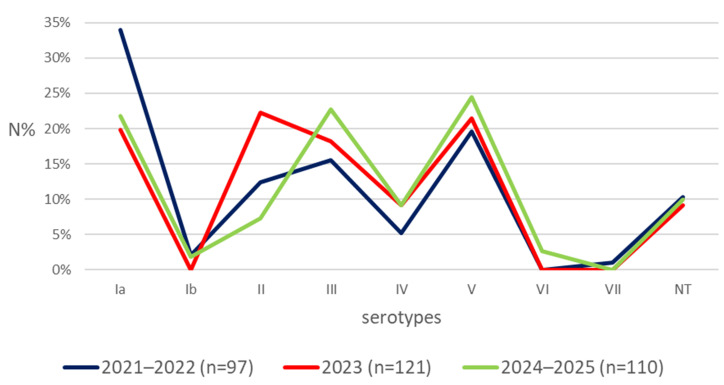
Frequency of serotypes during the respective years covering the study. Ia, Ib, II–VII—serotypes identified in GBS. NT—non-typeable. N%—percentage of total number of isolates identified in a given year.

Figure 2 demonstrates the fluctuation in the distribution of serotypes during the studied period (2021–2025). We categorized the isolates into three groups based on the year of isolation: 2021–2022 (97, 29.6%), 2023 (121, 36.9%), and 2024–2025 (110, 33.5%). The serotypes Ia, V, and III were the most prevalent in the years 2021–2022, while II, V, and Ia were predominant in 2023, and V, III, and Ia in 2024-2025. Statistical significance was observed in the distribution of serotype II (95% CI [0.062, 0.239], q < 0.05) between the groups 2023 and 2024–2025 (Table S2).

### 3.3. Distribution of Alp Among the Studied Strains

The vast majority of the analyzed isolates (95.1%) harboured the Alp gene determinants. The frequency of the genes encoding Alp was: *bca* (30.5%), *epsilon* (28.6%), *rib* (23.8%), and *alp 2/3* (12.2%). When comparing the frequency of these virulence factors according to the source of material from which GBS was isolated, statistical significance was not found between the distribution of the genes in the vaginal and extragenital samples (q > 0.05).

**Figure 3:**
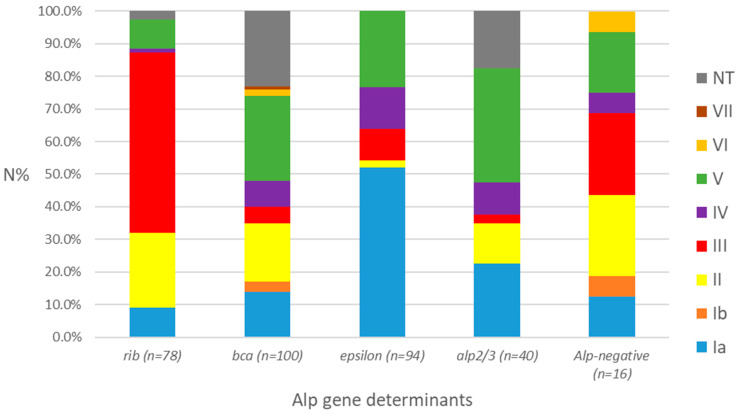
Distribution of the genes encoding Alp among serotypes. N%—percentage of identified serotypes in isolates that tested positive or negative for the Alp gene determinants. NT—non-typeable.

Regarding the distribution of these virulence factors in relation to the serotypes, we observed that *rib* was associated in 55.1% of cases with the most virulent serotype III, *bca* in 26.0% with serotype V, *epsilon* in 52.1% with serotype Ia, and *alp 2/3* in 35.0% with serotype V. Among the Alp-negative strains (4.9%), the most prevalent serotypes were III (31.3%), followed by II (25.0%), and V (18.8%) (Figure 3). There was a significant overall association between GBS serotypes and the *alp* genes (Table S3).

### 3.4. Examination of the Components Included in GBS Candidate Vaccines Among the Studied Isolates

All examined strains (*n* = 328) from both the vaginal and extra-vaginal groups were included in the calculation of the distribution of vaccine components. No exclusion criteria were established for patients concerning age, gender, or clinical presentation. The assessment of vaccine coverage was performed using the formula (number of isolates in the group/all examined strains) × 100.

Monovalent, bivalent, and trivalent candidate conjugate vaccines represented less than 50.0% of the examined strains, whereas pentavalent and hexavalent vaccines covered 266 (81.1%) and 292 (89.0%) of the isolates, respectively. Regarding the recombinant GBS-NN and GBS-NN2 vaccines, 178 (54.3%) and 134 (40.8%) of the examined isolates tested positive for their components, respectively. No statistical significance was found between the distribution of both conjugated and recombinant candidate vaccines in the vaginal and extra-vaginal samples (q > 0.05) (Table 1).

In comparison to the frequency of GBS-NN (*rib* + *bca*) and GBS-NN2 (*epsilon* + *alp2/3*) in relation to the serotypes, a statistically significant prevalence was observed between the distribution of serotypes II, III, and non-typeable isolates in the first vaccine and serotypes Ia and IV in the second vaccine (q < 0.05) (Table 2).

In comparison to our previous research on GBS isolates conducted between 2018 and 2019, statistical significance was observed for the *bca* gene and GBS-NN (*bca* + *rib*), which were prevalent in the earlier study (q < 0.05, 95% CI of the difference excludes zero) [36] (Table 3). A limitation of this partial comparison was that the previous research tested only serotype III and did not examine the components of GBS-NN2.

## 4. Discussion

The antibiotic prophylaxis in parturient women decreased the incidence of EOD by 86.0–89.0%. Disadvantages are limited effectiveness in preventing LOD, uncertain impact on disease in pregnant women, stillbirth, and preterm birth, as well as the emergence of antibiotic resistance, anaphylactic reactions to the administered medications, and alterations in the microbiota of the newborn. Some authors suggest that this prevention could potentially delay the onset of EOD, consequently leading to the development of LOD instead [4,37,38,39,40,41,42,43]. Furthermore, the spread of multidrug-resistant GBS strains poses significant challenges in treatment, especially for patients who are allergic to beta-lactams. Some isolates exhibit resistance to novel medications even before they are introduced into clinical practice [2,44]. The aforementioned challenges direct research efforts towards creating innovative methodologies, including the development of new vaccines [2,11,45]. During the vaccine development process, the potential candidates must meet the following key criteria: they should be highly conserved across different strains, perform essential functions, stimulate a protective immune response, elicit immune memory, and to ensure the safety of the patients [46,47]. Two main strategies at different stages of clinical trials have been prioritized during the development of the GBS vaccine: a vaccine based on capsular polysaccharides or protein antigens [25]. In the current study, approximately 70.0% of the examined isolates were obtained from vaginal samples, collected from pregnant and non-pregnant patients. Although there are no isolates from EOD or LOD, the primary target population for the GBS vaccines is pregnant women, thereby preventing possible transmission to the newborn. Furthermore, newborns may become colonized via non-maternal sources during both the perinatal and postpartum periods. Data indicates that vaccines could also be used among the adult non-pregnant population under certain settings [5,19]. A small number of studies examining the prevalence of GBS were conducted in Bulgaria in recent years, and there were no documented cases of invasive neonatal GBS strains in the past decade [36,48]. Additionally, in a multicenter study carried out in the adjacent country of Serbia between 2015 and 2020, the overall incidence of invasive GBS infections in infants was found to be lower than the global burden [49]. Consequently, although there is a notable limitation (the lack of neonatal GBS invasive isolates), our research provides valuable insights into GBS monitoring and can serve as a basis for future studies.

The capsule serves as an essential virulence factor in GBS. The geographical distribution of serotypes changes over time, with literature data indicating that serotypes I-V were identified in 98.0% of GBS colonising isolates worldwide, while VI-IX were mainly distributed in South-East Asia [50]. The predominant serotypes identified across various continents are: Europe III, Ia, and V; Africa—II, V, III, and Ia; Asia—Ia, III, and V; North America—Ia, V, and II; South America—Ia, II, and III [51,52,53,54,55]. Furthermore, certain serotypes were associated with specific clinical manifestations. Serotypes III and V were the most common among colonising strains, while serotype Ia was the most frequently isolated among GBS strains causing infections in mothers [50,56]. Serotype III is considered the most virulent and is the most common cause of late-onset meningitis and sepsis in newborns across all continents, followed by Ia and V. Serotypes Ia, V, and III are leading causes of EOD, as well as invasive infections in adults [5,20,57,58,59]. In discordance with the data provided, some studies reported an increase in the prevalence of serotype Ib in LOD and invasive infections among non-pregnant adult patients in China and South Asia, with this serotype being the most common in Japan [51,60,61]. In the current research, serotypes Ia, III, and V were detected with the highest frequency in all examined groups, thus confirming their leading role in Europe, with very similar prevalence data reported in Great Britain, Germany, Norway, and Switzerland [62,63,64,65,66]. Furthermore, our findings, although derived from a limited number of hospital-based sources, were consistent with global trends, highlighting a widespread prevalence of the six main serotypes (Ia–V) [50,52]. Based on the analysis of the serotype changes throughout the study period, we concluded that serotype II was predominant in the year 2023 compared to 2024–2025, underscoring the importance of investigating all serotypes and their associations. The observed shift from the predominance of Ia during 2021–2022 to a co-dominance with serotypes III and V in 2024–2025, although not significant, underlines the continuous fluctuations in serotype distribution. These aspects should be considered when using serotypes as the foundation for research and clinical trials.

The investigation and analysis of serotype prevalence and distribution is essential for the development of vaccines for immunising at-risk groups. In the initial clinical trials, the monovalent, bivalent, and trivalent vaccines were found to stimulate the production of IgG and trigger opsonophagocytic activity responses. In general, they were considered safe and well tolerated. However, following the introduction of the hexavalent vaccine, they were no longer tested due to the low coverage of circulating GBS strains [25,67]. The phase 2 clinical trials for the hexavalent vaccine, which assess the safety and immunogenicity in pregnant women, as well as the immune response when co-administered with the tetanus, diphtheria, and acellular pertussis vaccine, were completed in 2024. To date, no Phase III clinical trials have been conducted to evaluate any potential conjugate vaccine [11]. In the current research only pentavalent and hexavalent vaccines covered more than 80.0% of the examined isolates. The difference between them was 7.9%, attributed to serotype IV. Furthermore, considering the dynamics of serotype distribution among different countries, the phenomenon of capsular switching, and the presence of non-typeable strains, some authors proposed that the development of cross-reactive, gamma-irradiated, non-capsular whole-cell vaccines could be more effective [68]. Moreover, mutations in capsule synthesis or regulatory genes, along with hybridization among existing capsule types, may lead to the emergence of new serotypes [69]. The frequency of non-typeable strains correlates with the methodology employed, with sequencing-based methods demonstrating the lowest percentage of non-typeable strains. Although DNA sequencing is a more comprehensive method for characterizing pathogens than PCR, the latter remains faster, cost-effective, and easier to perform, despite its lower accuracy in GBS serotyping [49,63,70,71]. In the present study, 9.8% of the isolates were non-typeable. A similar or higher frequency has been documented in other studies [72,73,74,75].

According to literature data, over 95.0% of GBS isolates have at least one Alp protein. These surface proteins are essential for the bacteria to adhere to host epithelial cells. Additionally, they are important immunogenic determinants. Due to these characteristics, they were selected as candidates for vaccine development, with two recombinant vaccines GBS-NN and GBS-NN2 [15,18,76]. Protein vaccines could address serotype switching and provide a better target for vaccine development in comparison to polysaccharide vaccines. Currently, the combined recombinant GBS-NN/GBS-NN2 vaccine is in the second phase of clinical trials involving pregnant women. It exhibits good safety and immunogenicity profiles [11,19,25,45,77]. In the present study, we found that 95.1% of the studied strains possessed genes encoding an Alp protein. This corresponds to the widely accepted notion that these genetic determinants are ubiquitously distributed [16,18].

In a previous study in Bulgaria during 2018–2019, among 104 GBS strains, the genes *bca* and *rib* were found, with a rate of 51.0% and 22.1% (total 73.1%) [36]. In the present study, we report a significant decrease in the overall frequency of these genes, which is important due to their participation as components of the GBS-NN vaccine. The proteins included in GBS-NN2 were not examined in the previous study, limiting the ability to compare the findings with the current research. Nevertheless, the results for GBS-NN were statistically significant (q < 0.05), suggesting an epidemiological shift. Furthermore, the previous study only included serotype III, making it impossible to assess the evolution of serotypes. Despite these limitations, the importance of analyzing the available data serves as the basis for monitoring and provides a starting point for more comprehensive future comparisons.

Inconsistent distribution of the genes associated with Alp has been noted in data from other studies. In Spain, Argentina, Malaysia, South Korea, and Zimbabwe *bca* + *rib* predominated, whereas in Poland and the USA *epsilon* + *alp2/3* prevailed [32,78,79,80,81,82,83]. Regarding the distribution of these virulence factors in relation to the serotypes, we observed that certain serotypes were more frequently associated with specific Alp. This highlighted the importance of examining the components of the two protein vaccines to ensure maximum vaccine coverage [15].

Despite the limitations associated with the vaccines, such as their high cost and the lack of coverage for all GBS strains, in the year 2022, hexavalent and GBS-NN vaccine candidates received a status of great demand, along with enhanced support for the development by the European Medicines Agency (EMA) [77,84]. Our research provided crucial GBS surveillance data concerning all other serotypes, as well as GBS-NN/GBS-NN2 components.

Limitation statement: The limitations of the study included obtaining data from only two cities in Bulgaria. Although this study was conducted within a hospital-based setting, the collected samples were from three university hospitals that serve large regional cities and surrounding areas. Another limitation is the lack of sequencing-based methods; however, the techniques we applied were sufficient for our purpose. We observed a partial comparison with our previous research and noted the absence of isolates derived from invasive neonatal diseases. Although we did not have isolates from EOD or LOD, the primary target for GBS vaccines is pregnant women, as this helps prevent possible transmission to newborns. Additionally, cases of invasive neonatal GBS diseases have been reported to be rare in our region. The candidate vaccines may also be administered to non-pregnant adults, which is an aspect of our research.

## 5. Conclusions

This study investigated the distribution of important virulence factors among GBS isolates, which were included as components of candidate vaccines based on capsular polysaccharides or protein antigens. The prevalence of serotypes was consistent with data reported from Europe, thereby confirming the leading role of serotypes Ia, III, and V. Furthermore, serotypes I–V, which are part of the conjugated hexavalent vaccine, accounted for 89.0% of the examined isolates. Regarding protein-based vaccines GBS-NN/GBS-NN2, which covered 95.1% of the studied population, we observed a significant distribution of their components in relation to certain serotypes. Additionally, we noted a significant decrease in factors associated with GBS-NN, in comparison to our earlier study, which highlighted the importance of examining the components of the two protein vaccines in combination, which ensures optimal vaccine coverage. The data provided from GBS monitoring contributes to the continuous research focused on prophylactic and preventive strategies.

## Figures and Tables

**Table 1:** Distribution of vaccine candidates according to the material source.

Serotypes	Vaginal Samples (*n* = 227)	Extra-Vaginal Samples			Total Number (*n* = 328)	OR ^1^ [95% Cl ^2^]; *p*-Value ^3^; q-Value ^4^ (Vaginal/Extra-Vaginal Samples)
		Invasive (*n* = 54)	Non-Invasive (*n* = 47)	Total Extra-Vaginal (*n* = 101)		
Monovalent (III)	43 (18.9%)	11 (20.4%)	8 (17.0%)	19 (18.8%)	62 (18.9%)	1.01, [0.554, 1.836]; *p* = 1; q = 1
Bivalent (II and III)	75 (33.0%)	22 (40.7%)	12 (25.5%)	34 (33.7%)	109 (33.2%)	0.97, [0.592, 1.598]; *p* = 1; q = 1
Trivalent (Ia, Ib, and III)	106 (46.7%)	24 (44.4%)	17 (36.2%)	41 (40.6%)	147 (44.8%)	1.28, [0.797, 2.062]; *p* = 0.337; q = 0.786
Pentavalent (Ia, Ib, II, III, and V)	189 (83.3%)	45 (83.3%)	32 (68.1%)	77 (76.2%)	266 (81.1%)	1.55, [0.872, 2.757]; *p* = 0.169; q = 0.627
Hexavalent (Ia, Ib, II, III, IV, and V)	206 (90.7%)	48 (88.9%	38 (80.9%)	86 (85.1%)	292 (89.0%)	1.71, [0.842, 3.476]; *p* = 0.179; q = 0.627
GBS-NN (*rib + bca*)	123 (54.2%)	28 (51.9%)	27 (57.4%)	55 (54.5%)	178 (54.3%)	0.99, [0.618, 1.583]; *p* = 1; q = 1
GBS-NN2 (*epsilon + alp2/3*)	93 (41.0%)	22 (40.7%)	19 (40.4%)	41 (40.6%)	134 (40.8%)	1.02, [0.630, 1.636]; *p* = 1; q = 1

^1^ OR—odds ratio. ^2^ CI—confidence interval. ^3^ a *p*-value indicates the unadjusted significance levels for an individual test. ^4^ a q-value indicates a *p*-value that has been adjusted through the Benjamini–Hochberg procedure to control for the FDR during multiple comparisons [35]. Ia, Ib, II–VII—serotypes identified in GBS.

**Table 2:** Distribution of *rib* + *bca* and *epsilon* + *alp2/3* in relation to the GBS serotypes.

Serotypes	GBS-NN (*rib* + *bca*) (*n* = 178)	GBS-NN2 (*epsilon* + *alp2/3*) (*n* = 134)	OR ^1^ [95% Cl ^2^]; *p*-Value ^3^; q-Value ^4^ (GBS-NN/GBS-NN2)
Ia	21 (11.8%)	58 (43.3%)	0.18 [0.099-0.310]; *p* < 0.00001; **q < 0.00001**
Ib	3 (1.7%)	0	
II	36 (20.2%)	7 (5.2%)	4.60, [1.982, 10.682]; *p* = 0.0001; **q = 0.0002**
III	48 (27.0%)	10 (7.5%)	4.58 [2.219, 9.450]; *p* < 0.00001; **q < 0.00001**
IV	9 (5.1%)	16 (11.9%)	0.39 [0.168, 0.918]; *p* = 0.034; **q = 0.041**
V	33 (18.5%)	36 (26.9%)	0.62 [0.36, 1.06]; *p* = 0.098; q = 0.098
VI	2 (1.1%)	0	
VII	1 (0.6%)	0	
NT ^5^	25 (14.0%)	7 (5.2%)	2.96 [1.241, 7.080]; *p* = 0.0135; **q = 0.020**

^1^ OR—odds ratio. ^2^ CI—confidence interval. ^3^ a *p*-value indicates the unadjusted significance levels for an individual test. ^4^ a q-value indicates a *p*-value that has been adjusted through the Benjamini–Hochberg procedure to control for the FDR during multiple comparisons [35]. ^5^ NT—non-typeable. Ia, Ib, II–VII—serotypes identified in GBS.

**Table 3:** Comparison of the prevalence of serotype III and components of GBS-NN candidate vaccine between the study conducted from 2018 to 2019 and the current study.

	2018–2019 ^1^ (*n* = 104)	2021–2025 ^2^ (*n* = 328)	95% CI ^3^, *p*-Value ^4^; q-Value ^5^ (2018–2019/2021–2025)
Serotype III	14 (13.5%)	62 (18.9%)	[−0.133, 0.024]; *p* = 0.238; q = 0.238
*bca*	53 (51.0%)	100 (30.5%)	[0.097, 0.313]; *p* = 0.0002; **q = 0.0008**
*rib*	23 (22.1%)	78 (23.8%)	[−0.109, 0.076]; *p* = 0.791; q = 0.105
*Rib + bca* (GBS-NN)	76 (73.1%)	178 (54.3%)	[0.087, 0.289]; *p* = 0.0009; **q = 0.002**

^1^ Isolates from 2018 to 2019 [36]. ^2^ isolates from the current study. ^3^ CI—confidence interval. ^4^ a *p*-value indicates the unadjusted significance levels for an individual test. ^5^ a q-value indicates a *p*-value that has been adjusted through the Benjamini–Hochberg procedure to control for the FDR during multiple comparisons [35].

## Data Availability

The original contributions presented in this study are included in the article and Supplementary Material. Further inquiries can be directed to the corresponding authors.

## Supplementary Materials

The following supporting information can be downloaded at: https://www.mdpi.com/article/10.3390/cimb48040399/s1.

## Abbreviations

The following abbreviations are used in this manuscript:

GBS	Streptococcus group B
EOD	Early-onset disease
LOD	Late-onset disease
Alp	Alpha-like protein
EMA	European Medicines Agency
CP	Capsular polysaccharide
