# Supplementary material for: Gene Factors and Serotypes Related to Polysaccharide and Protein-Based Candidate Vaccines Among Streptococcus agalactiae Isolates
Source: Curr Issues Mol Biol. 2026 Apr 14;48(4):399. doi: 10.3390/cimb48040399 (PMC13114352; doi:10.3390/cimb48040399)
Supplement: Supplementary file 1 [file cimb-48-00399-s001.zip › cimb-4184399-supplementary.pdf]

Table S1. Primer sequences and amplification conditions for detection of GBS serotypes and the Alp gene determinants.

Genes	Primer sequence (5′→3′)	Product size (bp)	Annealing temperate (^{0}C)	Reference
Ia	F: GGTCAGACTGGATTAATGGTATGC<br>R: GTAGAAATAGCCTATATACGTTGAATGC	521 и 1826	61	[30]
Ib	F: TAAACGAGAATGGAATATCACAAACC<br>R: GAATTAACTTCAATCCCTAAACAATATCG	770	61	[30]
II	F: GCTTCAGTAAGTATTGTAAGACGATAG<br>R: TTCTCTAGGAAATCAAATAATTCTATAGGG	397	61	[30]
III	F: TCCGTACTACAACAGACTCATCC<br>R: AGTAACCGTCCATACATTCTATAAGC	1826	61	[30]
IV	F: GGTGGTAATCCTAAGAGTGAACTGT<br>R: CCTCCCCAATTTCGTCCATAATGGT	578	61	[30]
V	F: GAGGCCAATCAGTTGCACGTAA<br>R: AACCTTCTCCTTCACACTAATCCT	701	62	[30]
VI	F: GGACTTGAGATGGCAGAAGGTGAA<br>R: CTGTCGGACTATCCTGATGAATCTC	487	62	[30]
VII	F: CCTGGAGAGAACAATGTCCAGAT<br>R: GCTGGTCGTGATTTCTACACA	371	62	[30]
VIII	F: AGGTCAACCACTATATAGCGA<br>R: TCTTCAAATTCCGCTGACTT	282	62	[30]
IX	F: CTGTAATTGGAGGAATGTGGATCG<br>R: AATCATCTTCATAATTTATCTCCCATT	230	60	[31]
bca	F: TAACAGTTATGATACTTCACAGAC<br>R: ACGACTTTCTTCCGTCCACTTAGG	535	58	[32]
epsilon	F: TGATACTTCACAGACGAAACAACG<br>R: CCAGATACATTTTTTACTAAAGCGG	198	61	[33]
alp 2/3	F: TGATACTTCACAGACGAAACAACG<br>R: CACTCGGATTACTATAATATTTAGCAC	335	61	[33]
rib	F: TGATACTTTACAGACGAAACAACG<br>R: CATACTGAGCTTTTAAATCAGGTGA	296	60	[34]

F – forward primer, R – reverse primer

Table S2. Distribution of GBS serotypes for the period 2021-2025.

Serotypes	2021-2022 (n=97)	2023 (n=121)	2024-2025 (n=110)	95 % CI [1], p-value [2], and q-value [3] (2021-2022/2023)	95 % CI [1], p-value [2], and q-value [3] (2021-2022/2024-2025)	95 % CI [1], p-value [2], and q-value [3] (2023/2024-2025)
Ia	33 (34.0%)	24 (19.8%)	24 (21.8%)	[0.024, 0.260] p=0.020, q=0.190	[0.000, 0.244] p=0.061, q=0.356	[-0.125, 0.085] p=0.747, q=1
Ib	2 (2.0%)	0	2 (1.8%)		[-0.046; 0.056] p=1, q=1	
II	12 (12.4%)	27 (22.3%)	8 (7.3%)	[-0.198, 0,000] p=0.075, q=0.356	[-0.031; 0.133] p=0.245, q=0.729	[0.062, 0.239] p=0.002, **q=0.038**
III	15 (15.5%)	22 (18.2%)	25 (22.7%)	[-0.127, 0.072] p=0.717, q=1	[-0.179, 0.034] p=0.219, q=0.729	[-0.150, 0.059] p=0.417, q=0.792
IV	5 (5.2%)	11 (9.1%)	10 (9.1%)	[-0.107, 0.028] p=0.307, q=0.729	[-0.109, 0.030] p=0.298, q=0.729	[-0.074, 0.074] p=1, q=1
V	19 (19.6%)	26 (21.5%)	27 (24.5%)	[-0.127, 0.089] p=0.866, q=1	[-0.162, 0.063] p=0.408, q=0.792	[-0.139, 0.078] p=0.639, q=1
VI	0	0	3 (2.7%)			
VII	1 (1.0%)	0	0			
VIII	0	0	0			
IX	0	0	0			
NT [4]	10 (10.3%)	11 (9.1%)	11 (10.0%)	[-0.067, 0.092] p=0.820, q=1	[-0.079, 0.086] p=1, q=1	[-0.085, 0.067] p=0.827, q=1

[1] CI—confidence interval. [2] a p-value indicates the unadjusted significance levels for an individual test. [3] a q-value indicates a p-value that has been adjusted through the Benjamini-Hochberg procedure to control for the FDR during multiple comparisons [35]. [4] NT – non-typeable. Ia, Ib, II-IX –GBS serotypes.

Table S3. Associations between GBS serotypes and *alp* genes.

**Serotypes**	***rib*** (n=78)	**bca** (n=100)	***epsilon*** (n=94)	**Alp 2/3** (n=40)	**p-value** [1]
Ia	7 (9.0%)	14 (14.0%)	49 (52.1%)	9 (22.5%)	**p < 0.00001**
Ib	0	3 (3.0%)	0	0	
II	18 (23.1%)	18 (18.0%)	2 (2.0%)	5 (12.5%)	
III	43 (55.1%)	5 (5.0%)	9 (9.6%)	1 (2.5%)	
IV	1 (1.3%)	8 (8.0%)	12 (12.8%)	4 (10.0%)	
V	7 (9.0%)	26 (26.0%)	22 (23.4%)	14 (35.0%)	
VI	0	2 (2.0%)	0	0	
VII	0	1 (1.0%)	0	0	
NT [2]	2 (2.5%)	23 (23.0%)	0	7 (17.5%)	

[1] p-value calculated using Fisher's exact test with Monte Carlo simulation based on 10,000 replicates. a p-value ≤0.05 is considered statistically significant. [2] NT – non-typeable. Ia, Ib, II-VII – serotypes identified in GBS.
